# Homogeneous Diffusion Solid Model as a Realistic Approach to Describe Adsorption onto Materials with Different Geometries

**DOI:** 10.1186/s11671-016-1746-5

**Published:** 2016-12-12

**Authors:** E. Sabio, F. Zamora, C. M. González-García, B. Ledesma, A. Álvarez-Murillo, S. Román

**Affiliations:** 1 Departamento Física Aplicada, Escuela de Ingenieras Industriales, Universidad de Extremadura, 06006 Badajoz, Spain; 2 Departamento de Ingeniería Mecánica, Energética y de los Materiales, Escuela de Ingenieras Industriales, Universidad de Extremadura, 06006 Badajoz, Spain

**Keywords:** Activated carbon, PNP, Kinetics, HDSM, FEM

## Abstract

In this work, the adsorption kinetics of p-nitrophenol (PNP) onto several commercial activated carbons (ACs) with different textural and geometrical characteristics was studied. For this aim, a homogeneous diffusion solid model (HDSM) was used, which does take the adsorbent shape into account. The HDSM was solved by means of the finite element method (FEM) using the commercial software COMSOL. The different kinetic patterns observed in the experiments carried out can be described by the developed model, which shows that the sharp drop of adsorption rate observed in some samples is caused by the formation of a concentration wave. The model allows one to visualize the changes in concentration taking place in both liquid and solid phases, which enables us to link the kinetic behaviour with the main features of the carbon samples.

## Background

Phenolic derivate belong to a group of common chemicals which is characterized by the presence of the hydroxyl group (–OH) bonded directly to an aromatic hydrocarbon group. These compounds have a high antioxidant capacity, and their role in human health and disease is a subject of research. For this reason, there is an increasing interest in recovering of phenolic compounds from some industrial wastewaters by adsorption, such as olive mill wastewater [1, 2]. On the other hand, the presence of their even low concentration can be an obstacle to the use and/or reuse of water. Phenols cause unpleasant taste and odour of drinking water and can exert negative effects on different biological processes [3]. Most of these compounds are recognized as toxic carcinogens. Industrial sources of contaminants such as oil refineries, petrochemical, steel mills, coke oven plants, coal gas, synthetic resins, pharmaceuticals, paints, plywood industries, etc. generate large quantities of phenols [4].

Although many technologies have been implemented to recover or eliminate phenolic compounds, such as coagulation, foam flotation, filtration, ion exchange, aerobic and anaerobic treatment, electrolysis, etc., an adsorption process is still regarded as the best alternative based on its performance [5]. Adsorption techniques employing solid adsorbent have been widely used to remove certain classes of chemical pollutants from wastewater, such as phenolic compounds. Richard et al. [6] demonstrated that various adsorbents are available as the choices of adsorbent used to remove phenolic compounds from effluents via adsorption process, such as activated sludge, resins, modified alumina and activated carbon. Among all those aforementioned, activated carbons (ACs) are the most popular adsorbents that were used due their adsorption ability for relatively low-molecular-weight organic compounds such as phenols. In particular, the effectiveness of adsorption on activated carbons is mainly due their structural characteristics and their porous texture which gives them a large surface area [7].

Studies on the adsorption process of phenol and derivative compounds onto activated carbons have been carried out for some time and in fact still continue showing that the process presents some complexities. In this respect, the goal of achieving an optimum design for an adsorption process is an ambitious task for which a thoughtful knowledge of batch kinetics is necessary. Previous pieces of research on the kinetic analyses of batch adsorption processes have suggested the need of performing kinetic studies in which the adsorbent shape is considered, due to the major role that this feature can play during the process [8]. Although non-dimensional models (0D) are widely used to study the adsorption kinetics of phenol compounds, it should be noted that their resulting information is quite limited because geometry plays an important role in the mass transfer process between liquid and solid phases. The novelty of the article is the analysis of the effect of particle geometry on adsorption rate of phenol compounds. As far as we know, there are no previous studies of this effect reported in the literature.

In this work, the adsorption kinetics of the PNP was studied for several ACs with different geometrical and textural characteristics. For this purpose, a homogeneous diffusion solid model (HDSM) was applied by using the finite element method (FEM) with software COMSOL Multiphysics. In this model, the solid particle is considered to be surrounded by an external film offering a resistance to the mass transfer. Once the solute molecules enter into the pores of the solid particle, the model also assumes that the equilibrium adsorption is instantaneous and the adsorbed molecules move by diffusion. Accordingly, the equations describing the diffusion process inside the particle and the mass transfer through the external film are as follows:

1 $$ \frac{\partial q}{\partial t}+\nabla\ \cdotp\ \left(-D\ \cdotp\ \nabla \mathrm{q}\right)=0 $$

2 $$ D\ \cdotp\ \nabla q={k}_{\mathrm{f}}\ \cdotp\ \left({c}_{\mathrm{l}}-{c}_{\mathrm{s}}\right) $$

where *q* is the concentration of solute adsorbed (mg g^−1^), *D* the diffusion coefficient (m^2^ s^−1^), *k*_f_ the mass transfer coefficient in the external film (m s^−1^), *c*_l_ the solute concentration in the solution (mg l^−1^) and *c*_s_ the solute concentration at the solid surface (mg ^−1^).

## Methods

### Materials

Seven commercial activated carbons were used as adsorbents for the kinetic study of the PNP adsorption process: Darco activated carbon 20–40 mesh (Aldrich), granulated activated carbon (Scharlau), no. 3 QP activated carbon (Panreac), 2.5-mm granular activated carbon (Merck) (Albus), coconut shell activated carbon (Silcarbon), GCO 8 × 35 mesh (General Carbon Corporation) and MG1050 (MG, Chemivall). The samples were denoted as DA, SC, PA, AL, SIL, GCO and MG, respectively. The carbons were dried in oven at 105 °C for 12 h and kept in a desiccator until the samples reached room temperature.

Analytical-grade PNP (Sigma, purity > 99%) was used as an adsorbate. Water employed for solutions was distilled and deionized using a Milli-Q system (Millipore).

### Textural Characterization of Activated Carbons

The nitrogen adsorption isotherms at 77 K of the carbon samples were determined using a semi-automatic surface area analyser (AUTOSORB-1, Quantachrome). From the N_2_ adsorption data, the surface characteristic of ACs were obtained: (a) the specific surface area accessible to N_2_ determined by the BET equation (*S*_BET_) [9] and (*b*) the external or non-microporous surface (*S*_EXT_) by the *α*_S_ method [10] using non-porous solid proposed by Rodriguez-Reinoso et al. as reference [11].

Mercury porosimetry data (AUTOPORE 4900 IV, Micromeritics) were used to determine the mesopore (*V*_meP_) and macropore (*V*_maP_) volumes of the ACs.

The surface morphologies of the ACs were studied by scanning electron microscopy (SEM; Hitachi S-3600 N, Japan) to examine in details the particle morphology. The SEM samples were prepared by depositing about 50 mg of the AC on an aluminium stud covered with conductive adhesive carbon tapes and then coating with Rd-Pd for 1 min to prevent charging during observations. Imaging was done in a high vacuum mode at an accelerating voltage of 20 kV, using secondary electrons.

Geometrical particle characteristics were obtained using a digital processing toolbox. The activated carbon particles were photographed, and their surfaces were obtained using a geometrical pattern.

### Batch Experiments

Two series of batch-type experiments were carried out, equilibrium and kinetic.

#### Equilibrium Experiments

Equilibrium isotherms of PNP adsorption from aqueous solutions onto the activated carbons were performed by the batch method in an end-over-end stirrer at 11 rpm in an air thermostat bath at 20° ± 0.1 °C. For each equilibrium point, 50 mg of adsorbent was weighed in 20-ml stoppered tubes and 10 ml of a PNP solution of known initial concentration was added. The contact time was longer than that needed to reach equilibrium, as determined in previous experiments. After centrifugation, the supernatant solution was separated and the equilibrium concentration was evaluated spectroscopically with a Helios-α spectrophotometer (Unicam) at a wavelength of 225 nm, after dilution if that was necessary. The quantity adsorbed can be easily obtained from the change in the solution concentration before and after equilibrium with the carbons, according to the following:

3 $$ q=\frac{V\times \left({c}_0-c\right)}{m} $$

where *q* is the quantity adsorbed per unit of mass [mg g^−1^], *V* the solution volume [l], *c*_0_ the initial concentration [mg l^−1^], *c* the equilibrium concentration [mg l^−1^] and *m* the amount of activated carbon [g].

#### Kinetic Experiments

**Fig. 1:**
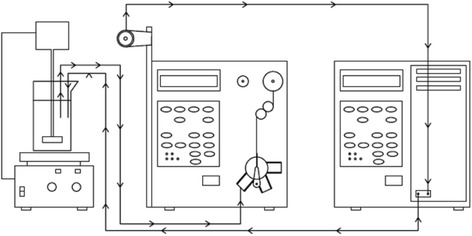
Experimental installation for the study of the PNP adsorption kinetics

PNP adsorption kinetics experiments were made with all activated carbons at temperature of 20 °C; for each run, different amounts of the activated carbon (0.1, 0.25 and 0.5 g) were added to 0.5 l of a PNP solution (80 mg l^−1^). The homogeneity of the different suspensions was assured by means of a shovel stirrer. The Solvent Delivery Module fed samples of the remnant suspension continuously to the measurement unit, which consisted of a UV/vis spectrophotometer at a wavelength of 225 nm. Once the solution concentration had been determined, the volume of sample was again incorporated to the solution-activated carbon system. The pH of the system was maintained in neutral conditions (pH = 7). Figure 1 shows a scheme of the experimental installation for studying the adsorption kinetics of PNP.

#### COMSOL Multiphysics

The resolution of the differential equations into partial derivatives was made using the software COMSOL Multiphysics. This programme creates an interactive environment for modelling and solving scientific and engineering problems based on partial differential equations. The Chemical Engineering Module was employed to model the transport and diffusion phenomena. “The adjusting parameters in COMSOL model (*D* and *k*_*f*_) were determined by fitting the experimental PNP adsorption kinetics data using the Levenberg-Marquardt algorithm.”

## Results and Discussion

### Activated Carbon Characterization

**Fig. 2:**
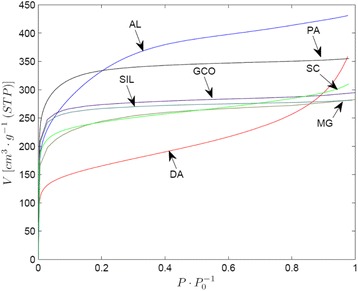
Adsorption nitrogen isotherms at 77 K

The N_2_ adsorption isotherms at 77 K for the ACs have been plotted in Fig. 2. Firstly, from the isotherms, it can be observed that the carbons AL and PA show a greater N_2_ adsorption capacity than the rest of the activated carbons.

From the shape of such isotherms, it may be stated that the samples PA, GCO, MG, SC and SIL present a high adsorption capacity at low relative pressures, which indicates the presence a well-developed microporous structure. After the initial section with a high slope, a near horizontal plateau is reached. These isotherms could be considered as type I of BDDT classification [10].

In the other hand, samples AL and DA approach the plateau more slowly and the values of adsorbed nitrogen increase steadily over the entire relative pressures range, which indicates a certain mesoporosity development. In the case of the DA sample, the amount of adsorbed nitrogen volume increases at high relative pressures, indicating a broader pore size distribution. In both cases, the isotherms would then be intermediate between types I and II of the referred classification.

Table 1 shows the textural characteristics of the ACs as determined from N_2_ adsorption data. It can be seen that AL, SIL, and PA shows a higher value of *S*_BET_. In general, all have a high microporosity, except the DA sample, with the SIL sample having the highest micropore volume. On the other hand, samples PA and specially DA show an external surface greater than that of the other ACs.

**Table 1:** Textural parameters of ACs obtained from N_2_ adsorption at 77 K, Hg porosimetry, and Langmuir parameters

Sample	*S*_BET_ (m^2^ g^−1^)	*S*_EXT_ (m^2^ g^−1^)	*V*_ma(Hg)_ (cm^3^ g^−1^)	*V*_me(Hg)_ (cm^3^ g^−1^)	*q*_m_ (mg g^−1^)	*b* (l mg^−1^)
SC	736	84	0.530	0.175	298.92	0.4136
DA	574	256	1.034	0.411	202.65	0.3250
AL	1155	84	0.544	0.265	389.79	0.1200
MG	788	50	0.334	0.113	265.18	0.0902
SIL	1057	17	0.352	0.085	341.19	0.0376
GCO	870	27	0.209	0.077	255.02	0.0422
PA	1181	70	0.247	0.127	327.63	0.0241

Table 1 also shows the collected textural parameters of the ACs obtained from the Hg porosimetry. It can be seen that the volume of macroporous is greater than that of mesoporous in all samples. DA shows a higher *V*_me_ and specially *V*_ma_. These results are consistent with the higher contribution of the external area associated to the former sample.

**Fig. 3:**
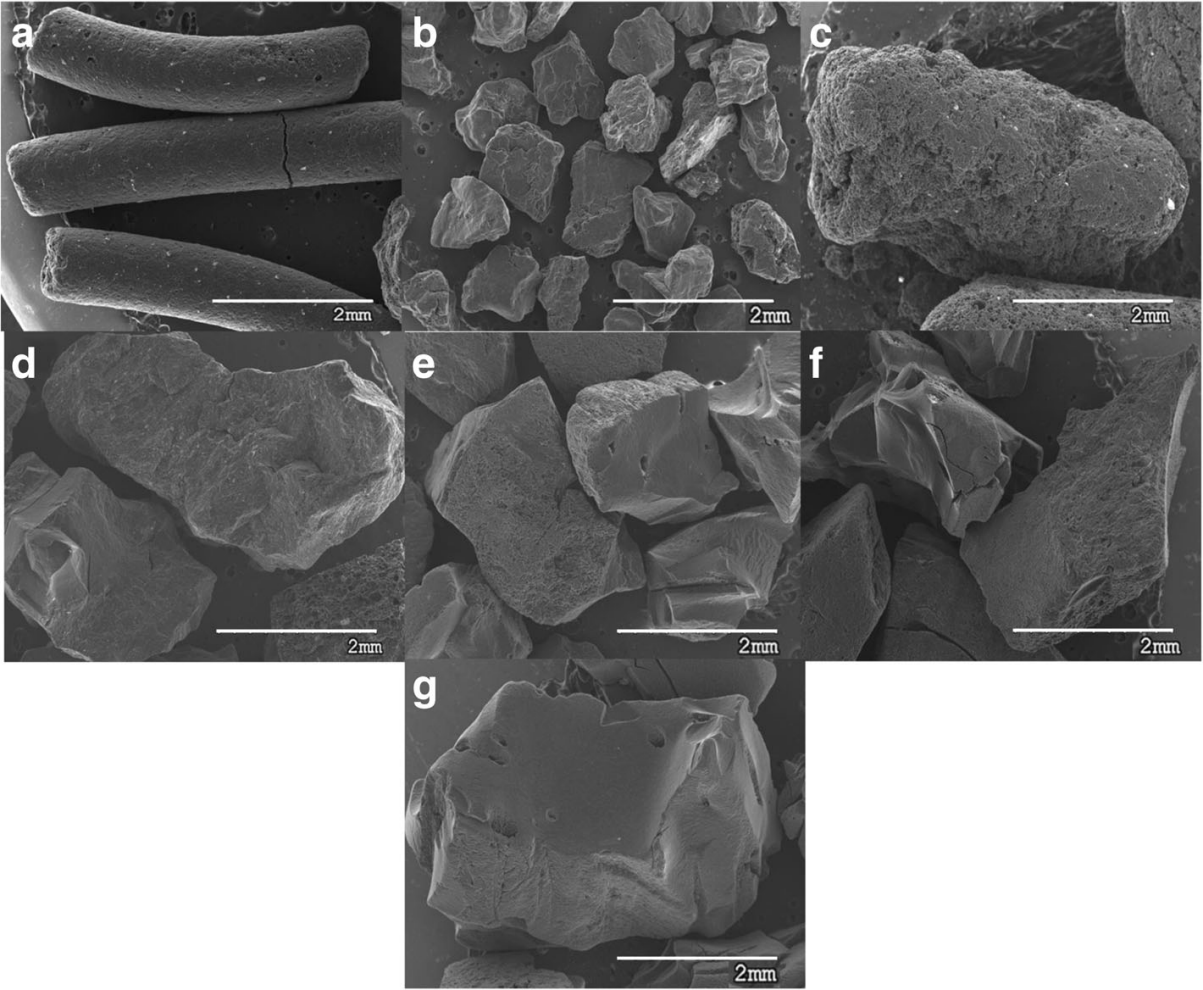
**a**–**g** SEM micrographs of the activated carbons. Magnification ×25

Particle size distribution and SEM analysis had enabled to determine geometrical characteristics of the particles and their particles surface/volume ratio (*S*/*V*). Figure 3a–g shows the SEM micrographs of the ACs and Table 2 their *S*/*V* value.

**Table 2:** Medium geometrical characteristics of the particles

	Radius (mm)	Height (mm)	Volume (mm^3^)	Surface (mm^2^)	S/V (mm^−1^)
SC	0.56	4.20	4.14	16.7	4.05
DA	0.45	–	0.38	2.54	6.67
AL	1.53	5.34	39.3	66.0	1.68
MG	0.76	–	1.84	7.26	3.95
SIL	0.89	–	2.95	9.95	3.37
GCO	1.05	–	4.85	13.9	2.86
PA	1.55	–	15.6	30.2	1.95

As it can be observed from Fig. 3, AL and SC samples have a cylindrical shape, while the remaining can be considered as a spherical shape. The differences in the geometrical shape of carbons make it necessary to use the diffusion equation with general geometrical conditions, with the boundary condition and the initial condition.

On the other hand, it can be observed from Table 3 that the DA and SC samples have a very high *S*/*V*, whereas the samples AL, GCO and PA have a low *S*/*V*.

**Table 3:** HDSM fitting results to the experimental values

		*D* (m^2^ s^−1^)	*k*_f_ (m^2^ s^−1^)	*R* ^2^
SC	0.1 g	4.7080e−12	9.013e−5	0.9542
	0.25 g	4.7080e−12	4.532e−5	0.9927
	0.5 g	4.7080e−12	3.015e−3	0.9932
DA	0.1 g	1.1997e−12	2.250e−5	0.9624
	0.25 g	1.1997e−12	1.511e−5	0.9924
	0.5 g	1.1997e−12	1.125e−5	0.9813
AL	0.1 g	1.7457e−11	5.121e−5	0.9997
	0.25 g	1.7457e−11	3.753e−5	0.9988
	0.5 g	1.7457e−11	3.254e−5	0.9944
MG	0.1 g	1.9762e−12	3.492e−5	0.9981
	0.25 g	1.9762e−12	2.9178e−5	0.9990
	0.5 g	1.9762e−12	2.513e−5	0.9949
SIL	0.1 g	2.6849e−12	3.531e−5	0.9995
	0.25 g	2.6849e−12	2.275e−5	0.9993
	0.5 g	2.6849e−12	1.421e−5	0.9978
GCO	0.1 g	5.8000e−12	4.012e−5	0.9978
	0.25 g	5.8000e−12	3.032e−5	0.9979
	0.5 g	5.8000e−12	2.051e−5	0.9931
PA	0.1 g	5.0102e−11	3.125e−5	0.9995
	0.25 g	5.0101e−11	2.875e−5	0.9986
	0.5 g	5.0101e−11	2.523e−5	0.9954

**Fig. 4:**
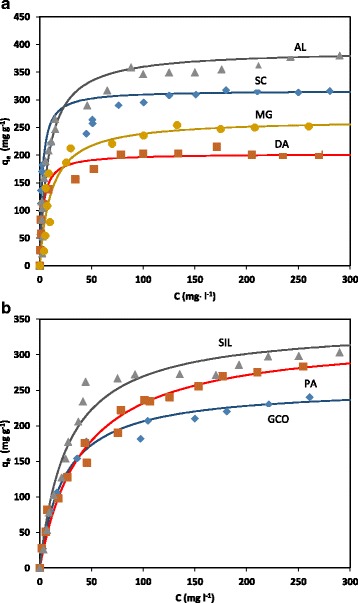
PNP adsorption isotherms for activated carbons and Langmuir fitting for **a** H isotherms and **b** L isotherms

The adsorption isotherms at 20 °C of PNP on the ACs are plotted in Fig. 4. As it is inferred from Fig. 4a, DA, AL, MG and SC p-nitrophenol adsorption isotherms can be classified as type H of the Giles classification [12]. In contrast, the GCO, PA and SIL ones can be classified as type L (Fig. 4b).

Experimental results were fitted to Langmuir equation and *q*_m_ (amount of solute adsorbed in the monolayer), and *b* (related to the energy of the solute-adsorbent interaction) parameters were obtained (Table 1).

The Langmuir equation results show that samples DA, AL, MG and SC have a higher value of *b* parameter than samples GCO, PA and SIL. It showed their affinity of the adsorbents towards the solute.

### Adsorption Kinetics

**Fig. 5:**
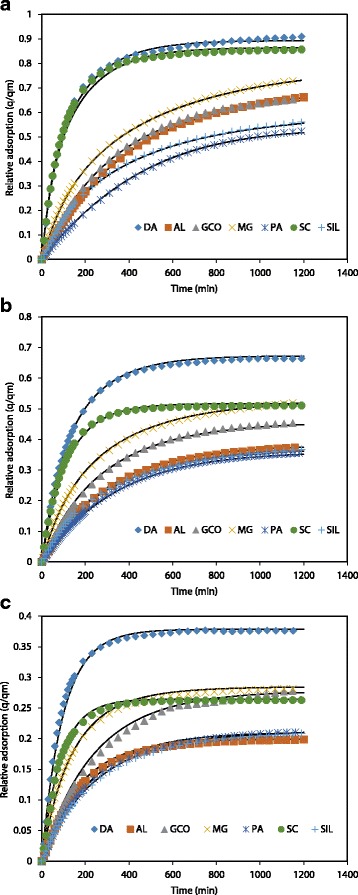
Adsorption kinetics for PNP onto the ACs for **a** 0.1, **b** 025 and **c** 0.5 g of solid, together with the fitting of the experimental data by HDSM

Figures 5a–c show the experimental PNP adsorption curves obtained for the seven adsorbent samples studied in the experiments carried out with 0.1, 0.25 and 0.5 g, respectively. From these figures, several relevant facts can be pointed out. First of all, all samples reduce their relative adsorption (*q*/*q*_m_) as the mass of solid rises from 0.1 to 0.5 g, being the effect especially marked in SC sample. Secondly, in the experiments carried out with 0.1 g, it can be observed that there are two main kinetic patterns. Samples DA and SC undergo a sharp initial adsorption, and then, at times around 200 min, the rate of adsorption decreases and, as a result, the curves tend to reach suddenly a plateau that correspond to the equilibrium conditions between liquid and solid phases. The rests of the samples adsorb at a slower rate, and the equilibrium plateau was not reached in 1200 min. Finally, as the mass of solid increases, the equilibrium plateau is better defined in samples DA and SC and tends to appear in the rest of the samples, which indicates that, in all cases, the equilibrium conditions would be achieved at shorter times as the mass of solid increase.

**Fig. 6:**
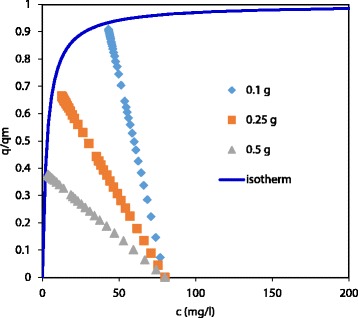
Langmuir isotherm of the adsorption of PNP onto DA sample and the operation lines of the experiments carried out with 0.1, 0.25 and 0.5 g

The fact that the amount of PNP adsorbed by the activated carbons decreased with an increasing adsorbent dosage has been described previously [13, 14]. This behaviour may be related to the different operation lines that present the experiments according to the mass of the carbon, that is, the successive liquid-solid concentrations taking place during the adsorption process, where the initial conditions are *c* = *c*_0_ and (*q*/*q*_m_) = 0 while the final conditions are those of the equilibrium (*c*_e_, (*q*/*q*_m_)_e_). Figure 6 shows a key example of the three operation lines occurring during adsorption of PNP in DA. It can be observed that as the amount of the adsorbent employed increased the operation line flat, which means that for a given solution concentration, *c*, the amount of adsorbed solute is lower, thus the driving force of adsorption will be higher. This fact would explain why, as the carbon dosage increases, the equilibrium is reached faster and with a lower value of (*q*/*q*_m_)_e_.

**Fig. 7:**
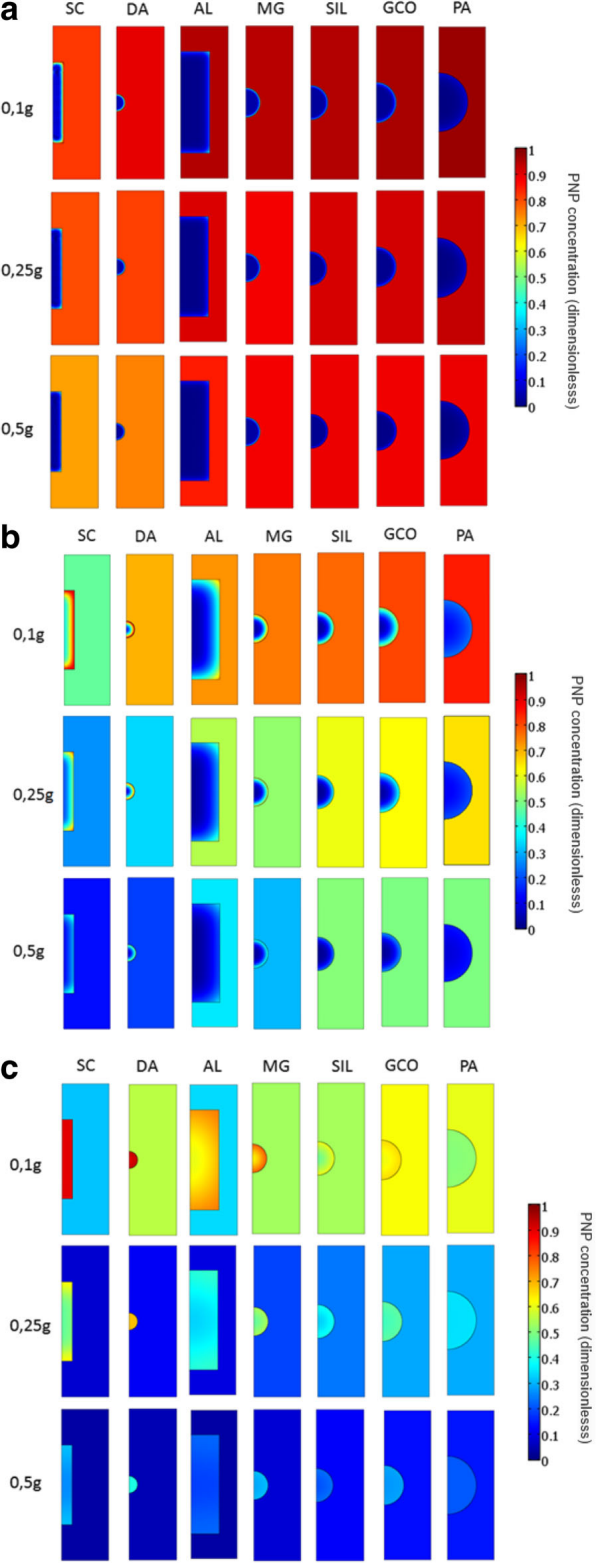
**a**–**c** 2D graphs for the concentrations of PNP in solid (*q*/*q*_m_) and liquid (*c*/*c*_0_) phases at 30, 200, and 1200 min

By using the 0D model, as the geometric factors are not considered, it is difficult to explain these features of the kinetic behaviour observed in the experiments carried out (Figs. 7a–c). In order to obtain information taking into account the role play by the geometry of the systems, the experimental data have been fitted to a HDSM, where the fitting parameters were the diffusion coefficient inside the particle (*D*) and the film resistance coefficient (*k*_f_).

The adjusting parameters values obtained are summarized in Table 3. The high values of the corresponding *R* ^2^ coefficient put in evidence the good agreement between the experimental and theoretical results, which indicates that the model describes properly the kinetics of the PNP adsorption for the studied samples, as can be observed in Fig. 5a–c. The good fit endows further analysis in providing a true picture of the adsorption process.

By applying the model, the maximum adsorption rate (*r*_ads_max_) has been calculated and the resulting data are presented in Table 4. This parameter can be fitted to the following linear equation:

**Table 4:** Maximum adsorption rate and fitting parameter of equation [4]

Mass of carbon (g)	Sample	*r*_ads_max_ (l/min)	*β*_1_	*β*_2_	*R* ^2^
0.1	SC	9.10e−03	0.242	0.816	0.980
	DA	7.98e−03			
	AL	1.76e−03			
	MG	2.99e−03			
	SIL	2.22e−03			
	GCO	2.16e−03			
	PA	1.16e−03			
0.25	SC	4.55e−03	0.515	0.579	0.986
	DA	5.32e−03			
	AL	1.32e−03			
	MG	2.57e−03			
	SIL	1.45e−03			
	GCO	1.85e−03			
	PA	1.06e−03			
0.5	SC	3.04e−03	0.568	0.507	0.954
	DA	3.98e−03			
	AL	1.14e−03			
	MG	2.13e−03			
	SIL	8.98e−04			
	GCO	1.24e−03			
	PA	9.23e−04			

4 $$ {r}_{\mathrm{ads}\_ \max }={\beta}_1\times \left(\frac{S}{V}\right)+{\beta}_2\times b $$

where *S*/*V* and *b* are the surface/volume ratio of the particle and *b* Langmuir parameter, respectively. On the other hand, *β*_1_ and *β*_2_ are the standardized linear coefficients. Table 4 shows the values of *β*_*1*_, *β*_*2*_ and *R* ^2^ obtained for the experiments carried out at 0.1, 0.2 and 0.5 g of solid mass.

It can be observed that, as the mass of carbon increases, *β*1 and *β*2 increases and decreases, respectively. This means that the solute accessibility into the carbon is getting a more relevant role in determining the adsorption kinetics while the solute-adsorbent interactions are losing prevalence. It should be taken into account that all the experiments were carried out by using 0.5 l of a PNP solution with a initial concentration equal to 80 mg l^-1^. Thus, an increment in carbon mass gives rise to larger entrance surface into the carbon, for the same amount of solute. As a result the parameter *S/V* would become more important, subtracting relevance to *b*.

According to the *R* ^2^ values in this table, it can be observed that, in the three set of experiments carried out, the linear models explain more than 95% of the variance found in the maximum adsorption rate data. This put in evidence that both *S*/*V* and *b* variables play a main role in the kinetics of the PNP adsorption. This fact is interesting because one could expect that variables such as *S*_BET_ and *q*_m_ would have a important influence on the adsorption kinetics. According to our results, adsorbents with particles having a high external surface in relation with their volume and where solute-solid interactions are strong would adsorb the solute very fast and conversely. This result is interesting from a practical point of view in choosing an appropriated adsorbent for a given application.

From the above results, it is obvious that geometry has an important influence in the adsorption process. One of the main advantages of using dimensional models is that they allow us to continuously visualize what is happening in both solid and liquid phases, facilitating the understanding of the overall mass transfer process. Figure 7a–c shows the 2D graphs of relative adsorption (*q/q*_m_) into the particle and relative remaining concentration in the solution (*c/c*_0_) for 30, 200 and 1200 min. The colour of the scale gives the quantitative values for the concentrations of PNP in solid (*q/q*_m_) and liquid (*c/c*_0_) phases. These graphs can be regarded as movie frames, at the given times, of the mass transfer process between both phases.

The graph at 30 min shows that the SC and DA samples adsorb PNP very fast in the initial stages, which results in a drop in the concentration of PNP in the solution (*c*/*c*_0_) while the carbon particles seems to be very far from the saturation conditions (Fig. 7a). The descent in (*c/c*_0_) is higher as the mass of carbon increased, as expected.

**Fig. 8:**
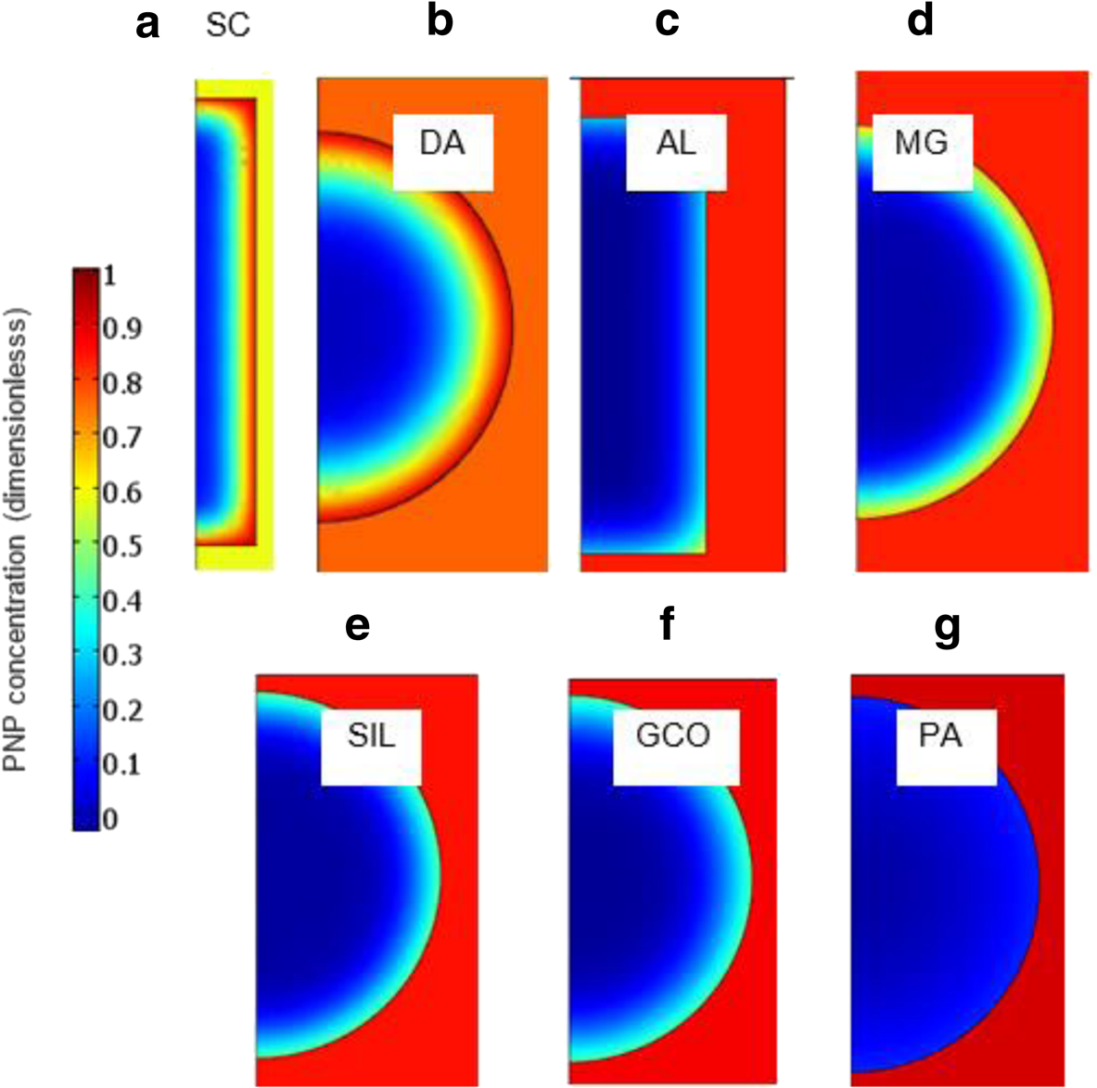
Detail of particle PNP concentration (*q*/*q*_m_) at 200 min and 0.1 g for the ACs studied

At 200 min, there is a clear PNP concentration wave in SC and DA particles (Fig. 7b). That is, the outer part of the SC and DA particles shows a sharp negative gradient of PNP concentration, beyond which the solute concentration is very low (Fig. 8 shows a detail of particle concentration). Probably, the formation of this wave is a result of the combination of two effects in the SC and DA samples: the fast initial adsorption and the low diffusion coefficient (*D*). The low *D* value means that there is a slow motion of the adsorbed solute inside the particles, which avoids an even PNP distribution in the solid. As a result, the surface of the SC and DA particles shows a very high PNP concentration, near to saturation (*q*/*q*_m_~1), hindering the liquid-solid transference of the solute. This wave of concentration would explain the fast drop of the adsorption rate observed in the SC and DA samples around 100–200 min. In the other samples, this wave is starting and has a small concentration gradient, thus the adsorption impediment resulting from an increases of PNP concentration at the particle surface is relatively low (Fig. 8).

At 1200 min (Fig. 7), it can be observed that, in all cases, the solid particles have a homogeneous concentration, which would indicate that the equilibrium between liquid and solid phases has almost been achieved for all samples, no matter what the solid mass used. This is in agreement with a behaviour observed in Fig. 5, where the adsorption curves tend to reach a plateau.

## Conclusions

We have described a study of the PNP adsorption kinetics in seven activated carbons. The adsorbents were characterized in terms of their textural, liquid equilibrium adsorption of PNP and geometric characteristics. The resulting kinetic experimental data were modelled by using a HDSM.

The following conclusions can be drawn from the results of the study:

1. The seven samples showed very different textural, geometric, and adsorbent properties. The SC and AL sample particles were cylindrical while the rest of the samples had spherical particles. On the other hand, samples PA, SIL, and AL presented the largest internal surface (*S*_BET_) and DA the smallest. As a result, the former samples had the highest *q*_m_ values while DA showed the smaller. As far as the *S/V* ratio and *b* are concerned, SC and DA showed the highest values for these variables.
2. The dimensional model developed (HDSM) fits properly the experimental results of the kinetics adsorption of PNP onto the ACs studied. The model allows the kinetic patterns observed to be described in terms of the adsorption process taking place in the solid particle. Accordingly, the fast initial adsorption rate in the SC and DA samples drops markedly around 200 min as a result of the formation of a sharp wave of concentration, with negative gradient, in the outer part of the ACs particles.
3. The rate of the adsorption process depends mainly on the geometric parameters of the particle (*S*/*V*) and on the strength of the solute-solid interactions (*b*).
